# Comparing the Epidermal Growth Factor Interaction with Four Different Cell Lines: Intriguing Effects Imply Strong Dependency of Cellular Context

**DOI:** 10.1371/journal.pone.0016536

**Published:** 2011-01-31

**Authors:** Hanna Björkelund, Lars Gedda, Karl Andersson

**Affiliations:** 1 Ridgeview Instruments AB, Uppsala, Sweden; 2 Department of Oncology, Radiology and Clinical Immunology, Biomedical Radiation Sciences, Uppsala University, Uppsala, Sweden; University of South Florida College of Medicine, United States of America

## Abstract

The interaction of the epidermal growth factor (EGF) with its receptor (EGFR) is known to be complex, and the common over-expression of EGF receptor family members in a multitude of tumors makes it important to decipher this interaction and the following signaling pathways. We have investigated the affinity and kinetics of ^125^I-EGF binding to EGFR in four human tumor cell lines, each using four culturing conditions, in real time by use of LigandTracer®.

Highly repeatable and precise measurements show that the overall apparent affinity of the ^125^I-EGF – EGFR interaction is greatly dependent on cell line at normal culturing conditions, ranging from K_D_≈200 pM on SKBR3 cells to K_D_≈8 nM on A431 cells. The ^125^I-EGF – EGFR binding curves (irrespective of cell line) have strong signs of multiple simultaneous interactions. Furthermore, for the cell lines A431 and SKOV3, gefitinib treatment increases the ^125^I-EGF - EGFR affinity, in particular when the cells are starved. The ^125^I-EGF - EGFR interaction on cell line U343 is sensitive to starvation while as on SKBR3 it is insensitive to gefitinib and starvation.

The intriguing pattern of the binding characteristics proves that the cellular context is important when deciphering how EGF interacts with EGFR. From a general perspective, care is advisable when generalizing ligand-receptor interaction results across multiple cell-lines.

## Introduction

Cells are complex units with a heterogeneous surface. It is therefore likely that a ligand interacting with a cell binds to more than one receptor. These receptors may be members of the same receptor family or even the same receptor in different conformations. Nevertheless, the common discussion about biomolecular interactions tends to be simplistic. It is often assumed that most interactions are so called 1∶1 interactions, with one monovalent ligand binding to one specific target [1].

Signs of heterogeneity have been described for the epidermal growth factor (EGF) interacting with the EGF receptor (EGFR). Previous results indicate two receptor populations: one binding with high affinity (10–100 pM) and one with low affinity (1–10 nM) [2]. The epidermal growth factor receptor family consists of four members: EGFR (HER1/ErbB1), HER2, HER3 and HER4. These receptors are known to dimerize, with themselves (homodimers) or with other members of the EGF receptor family (heterodimers). To what extent the dimerization occurs and its correlation to ligand binding and downstream signaling has been discussed for many years and is not yet fully understood. A common opinion is that the dimerization requires conformational changes triggered by the binding of EGF [3], [4], [5], although some claim that there can be dimers on the cell surface even without bound EGF [6], [7]. In addition to numerous experimental procedures, the mechanism of the EGF-EGFR interaction has been investigated with advanced kinetic modeling tools as well [8].

Atypical expression or activity of EGFR is present in numerous kinds of cancer [9]. Therefore, the EGF receptor family has become an important target for cancer therapy. One example is gefitinib (also denoted IRESSA™ or ZD1839), designed for blocking downstream signaling by tyrosine kinase inhibition on non-small cell lung cancer [10]. The result is inhibited growth, but the response varies and the majority of patients show no response to the treatment [11]. Why some cell lines and patients are resistant to gefitinib treatment remains unclear, although several hypotheses are mentioned in the literature. For example, mutations in the intracellular domain linked to internalization deficiencies are overrepresented in gefitinib sensitive cell lines, even though gefitinib binds to the mutated EGFR with the same affinity [12]. Moasser et. al. [13] discusses the link between HER2 expression and gefitinib sensitivity. Gefitinib binding may affect the extracellular part as well, with an apparent increase in EGF uptake, as observed both in gefitinib sensitive cells and in cells where growth rate is not affected [14].

The aim of this study was to provide new information on the intricate interaction pattern of EGF and EGFR by comparing the kinetics and the affinity of the ^125^I-EGF-EGFR interaction in four cancer cell lines. We searched for signs of multiple interactions occurring simultaneously and compared the affinity. The cells were exposed to four different treatments and gefitinib sensitivity and impact of starvation of the cell lines were studied.

### Theory

Since the reversible 1∶1 interaction model is the general choice when discussing biological interactions, we will start with the same assumption. It can be described by
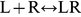
(1)where free ligand L binds to the receptor R to form the complex LR. The formation over time can be described with the differential equation
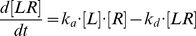
(2)where k_a_ (M^−1^s^−1^) is the association rate constant and k_d_ (s^−1^) is the dissociation rate constant describing the formation and dissociation of the complex. The amount of complex can be rewritten as [15]

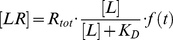
(3)where R_tot_ is the total amount of receptors (bound and unbound) and K_D_ is the equilibrium dissociation constant (often denoted affinity). K_D_ is in turn the ratio between k_d_ and k_a_,
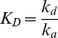
(4)


Equation (3) can be utilized when calculating the amount of receptors bound at a specific concentration of the ligand L. For example, if using a ligand concentration of 0.1×K_D_, 1×K_D_ or 10×K_D_, the amount of bound receptors at equilibrium will be 10%, 50% or 91% respectively.

At steady-state, f(t) = 1. In a biological context, the effect of an interaction often occur long before equilibrium has been reached (because time to equilibrium can be many hours or days) and the time for the effect to remain is often due to the dissociation time [16]. Therefore, it is important to study how fast the ligand associate and dissociate to its receptor. In these cases f(t), i.e. how the interaction proceeds over time, can be described by
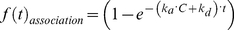
(5)

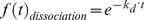
(6)


Equations (5) and (6) describe how the affinity and the curvature of a real-time binding trace are related. Equation (6) can be translated as “the lower the k_d_, the slower the dissociation”, which combined with (4) means that a slow dissociation also is a sign of a low K_D_, i.e. a high affinity. Equation (5) is more difficult to grasp, as both k_a_ and k_d_ are involved, but in general a higher affinity will result in less curvature and longer time to equilibrium.

## Results and Discussion

### Interaction analysis of ^125^I-EGF – EGFR using four cell lines

Data from stepwise titration affinity experiments was used to estimate the equilibrium dissociation constant K_D_ of the ^125^I-EGF – EGFR interaction. The signal levels from the end of each ^125^I-EGF incubation, representing approximately steady-state, were analyzed using a non-linear regression model describing a 1∶1 interaction. Calculations of K_D_ were made using data from two titration measurements for each cell line. A 1∶1 interaction model is not sufficient to describe the system, as ^125^I-EGF seems to bind to the cells in a multiple manner (see below), but the model was still chosen to provide results comparable with common saturation curve analysis in literature.

**Figure 1:**
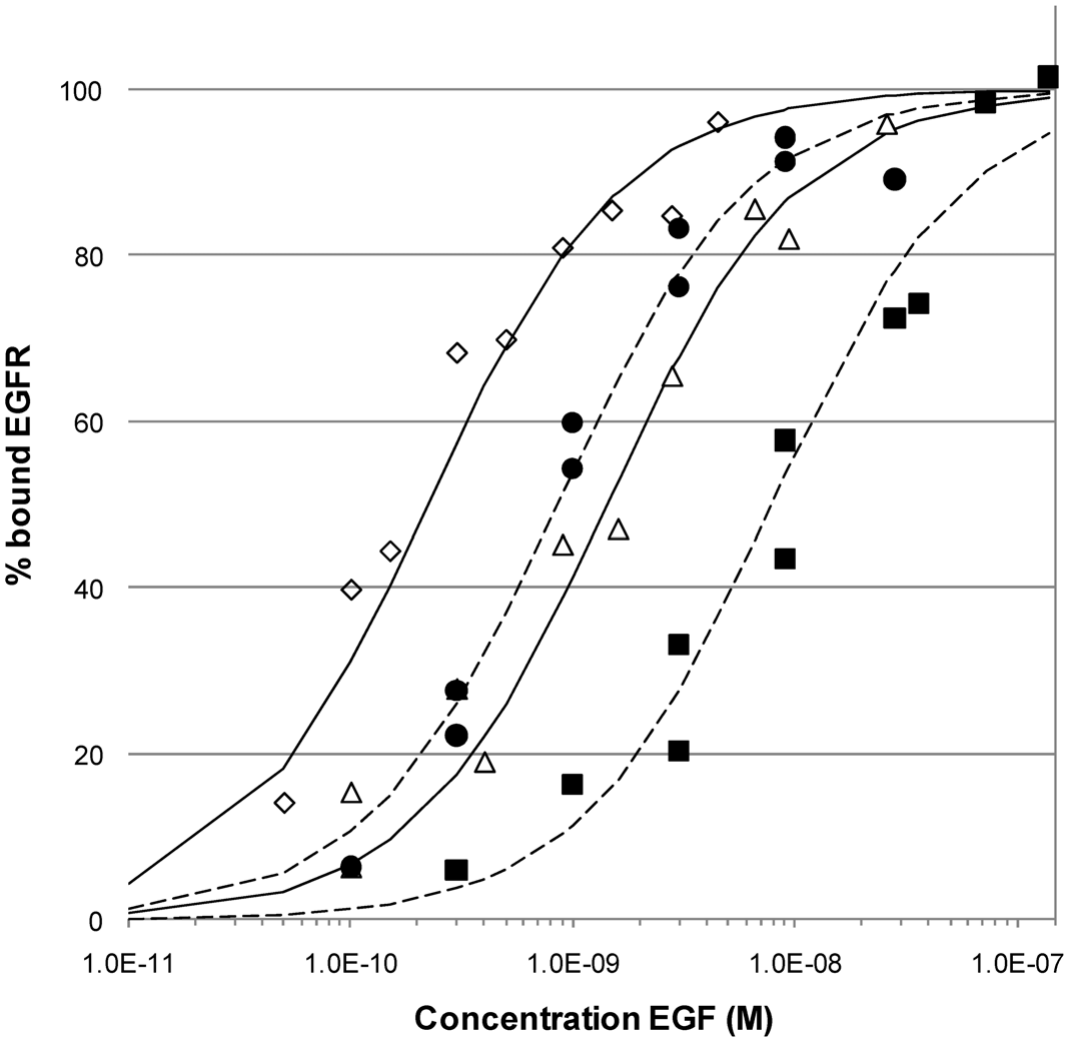
Saturation measurements showing great differences in affinity. Saturation measurements and non-linear regression 1∶1 fits for the ^125^I-EGF – EGFR interaction on SKBR3 cells (open diamonds, solid line), SKOV3 cells (filled circles, dashed line), U343 cells (open triangles, solid line) and A431 cells (filled squares, dashed line). The data indicates that the affinity of the interaction varies between the four cell lines, from 0.2 nM for SKBR3 to 8 nM for A431.

Figure 1 shows the signal versus concentration graphs for each cell line, together with the calculated affinity fits. The estimated affinity varied largely: The ^125^I-EGF – SKBR3 interaction had the highest affinity, with a K_D_ value of approximately 0.2 nM. ^125^I-EGF binds to SKOV3 and U343 cells with an intermediate strength, with K_D_ values of approximately 0.9 and 1.4 nM respectively. A431 cells show the weakest interaction, with a K_D_ of as much as approximately 8 nM. The difference of a factor 40 between the strongest and the weakest interaction came as a surprise, as the ^125^I-EGF – EGFR interaction was expected to be the same no matter the cell. To our best knowledge, there are no known mutations on the extracellular part of EGFR in any of the four cell lines that could affect the affinity.

**Figure 2:**
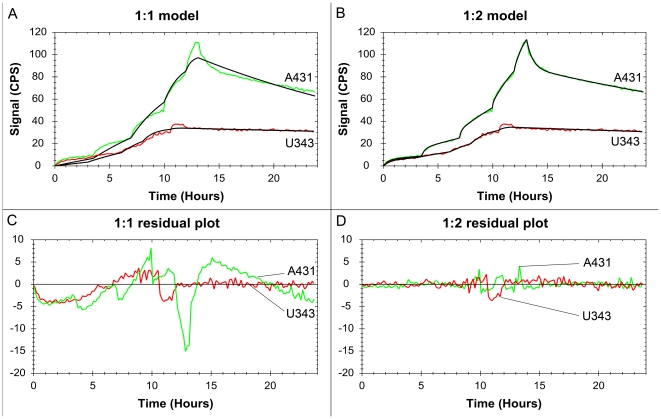
Fitting binding curves to 1∶1 and 1∶2 interaction models. (A) 1∶1 and (B) 1∶2 interaction models (black) fitted to titration data of ^125^I-EGF binding to A431(green) and U343 (red) cultured cells. The deviations of the fitted models from the data are described in residual plots for the (C) 1∶1 and (D) 1∶2 interaction models. The 1∶1 interaction model fits the data poorly.

When evaluating the kinetics of the ^125^I-EGF – EGFR interaction the data showed signs of multiple interactions taking place, observed as binding curvatures different from ordinary 1∶1 interactions. This was concluded by fitting the binding curves from the affinity titration to models describing one monovalent ligand (^125^I-EGF) binding to either one or two independent receptors on the cell with different kinetic parameters. Representative data from one A431 and one U343 affinity measurement is depicted in figure 2. Residuals plot were calculated, describing the differences between the measured data and the calculated fits. It is clear that the 1∶2 model is superior in fitting the data, which can be observed in the signal versus time plots (Fig. 2A and B, black curves) as well as in the residual plots where the differences between fitted and measured data are greater for the 1∶1 model than for the 1∶2 model (Fig. 2C and D). The same observations were made for SKBR3 and SKOV3 (data not shown). This indicates that there are at least two, maybe more, interactions occurring simultaneously, and it is in line with the description of a low affinity and a high affinity population of EGFRs [2]. The small fraction of high affinity EGFR is likely the reason to why its impact is seldom accounted for, even though there are studies proposing that the high-affinity receptors are the primary mediators of the EGFR signaling [17]. Worth pointing out is that the effect of the populations on the interaction measurement will be different depending on the concentration used. At low concentration the high affinity interaction will be dominating. At higher concentration the high affinity receptor may already be saturated, which means that any signal increase is due to low affinity interactions. This makes it essential to study a wide concentration span to ensure an accurate analysis of any multiple interactions.

One possible explanation for the large differences in affinity and the multiple simultaneous interactions is the presence of EGFR homo- and hetero-dimers. Varying EGFR and HER2 receptor expression levels could lead to different proportions in the homo/hetero-dimer population and this could play a significant role for the interaction pattern and apparent affinity for a particular cell line. A431 and U343 over-express EGFR (2×10^6^ receptors/cell and 5×10^5^ receptors/cell respectively) [18] and have a lower HER2 expression [13], [19]. SKOV3 and SKBR3 have a large HER2 population and a lower EGFR expression [13].

### The effect of starvation and gefitinib on the ^125^I-EGF – EGFR interaction

The uptake and retention of ^125^I-EGF to the cultured cell lines A431, U343, SKOV3 and SKBR3 were monitored in real-time using LigandTracer. The cells were exposed to four different treatments (control, starvation, gefitinib and gefitinib +starvation) prior to and during the measurements. Uptake measurement consisted of a two-step incubation, with pairs of concentrations chosen to receive a clear increase in signal by the second addition of ^125^I-EGF. As the affinity study proved that the nature of the ^125^I-EGF – EGFR interaction varies greatly between the cell lines, the concentration pairs for the measurement had to be chosen differently for the cell lines to obtain comparable results for the treatment effect analysis.

**Figure 3:**
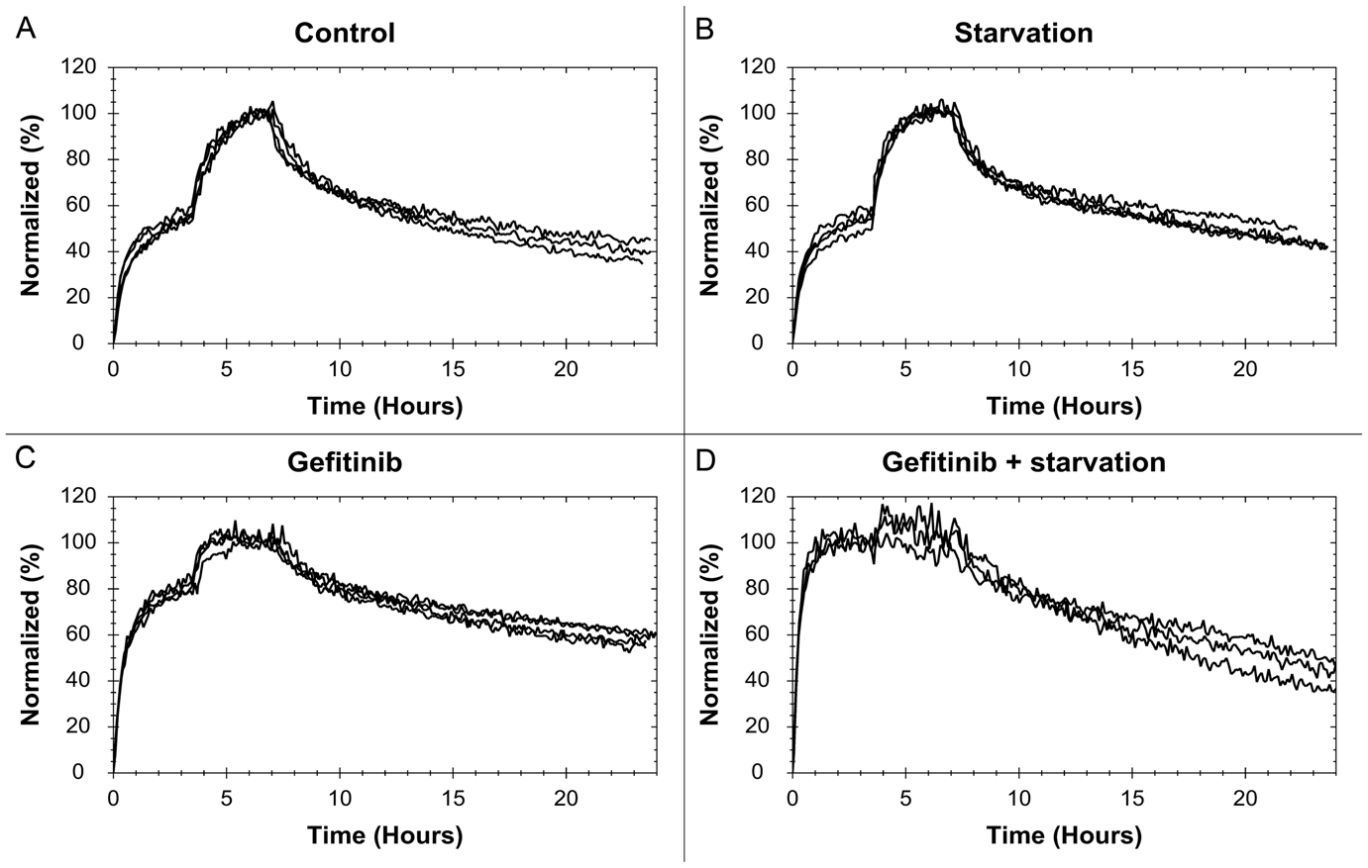
The effect of gefitinib and starvation on the ^125^I-EGF-EGFR interaction on A431 cells. Binding curves from the ^125^I-EGF-A431 measurements, with three-four replicates for each treatment: (A) control, (B) starvation, (C) gefitinib and (D) gefitinib +starvation.

**Figure 4:**
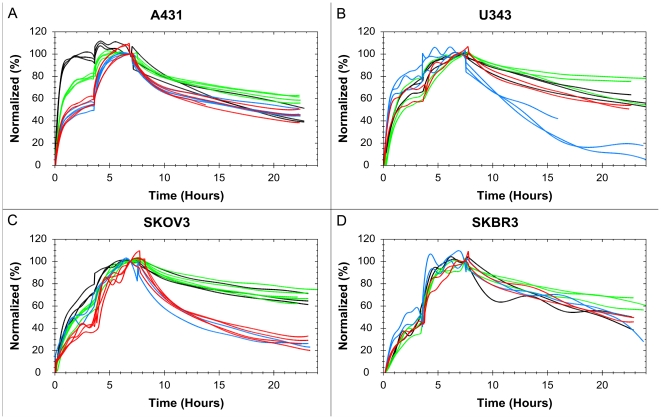
The effect of gefitinib and starvation on the ^125^I-EGF-EGFR interaction on four tumor cell lines. ^125^I-EGF binding to A431 (A), U343 (B), SKOV3 (C) and SKBR3 (D), using four different treatments: control (red), starvation (blue), 1 µM gefitinib (green) and 1 µM gefitinib + starvation (black). Data shows that the affinity of the EGF-EGFR interaction on A431 and SKOV3 is affected by gefitinib exposure and that the effect is boosted in starvation medium. U343 cells are sensitive to starvation and possible to some extent to gefitinib (slower dissociation). SKBR3 cells seem unaffected by all treatments.

Normalized interaction data for each treatment of A431 shows good reproducibility for the method (Fig. 3). To enhance visibility, noise reduced results for all cells are found in figure 4. The curves have been normalized to have a 100% binding at the end of incubation 2 to make comparisons possible. This is not equivalent to having a saturation of the receptors at that point.

The ^125^I-EGF – A431 interaction is clearly affected by the presence of gefitinib, in particular in serum free medium. Gefitinib treated cells have a smaller difference in signal level between the first and second concentration (Fig. 4A), thus the first concentration is closer to saturating the receptor population. This means that there is a shift in the [L]:K_D_ ratio and consequently gefitinib treatment increases the affinity of the interaction, possibly as much as 10 times for the gefitinib +starvation cells. A higher affinity is also observed in the curvature itself, with a slower dissociation.

U343, which does not respond to gefitinib treatment in growth rate studies [14], may be insensitive to gefitinib regarding the ^125^I-EGF -EGFR interaction as well. There are weak signs of an increased affinity when exposed to gefitinib (Fig. 4B), observed as a somewhat slower dissociation. For the starved cells the dissociation rate is clearly higher, indicating a lower affinity. The results from the starvation studies (+/−gefitinib) were noisy and in some cases the cells detached. Starved U343 cells seem more unstable and binds ^125^I-EGF with a lower affinity, which is relevant from a drug design perspective as there is often limitations in proper nutrition inside solid tumors [20].

Gefitinib clearly affects the ^125^I-EGF interaction with SKOV3, although the effect is not as large as for A431 (Fig. 4C). Starvation has no obvious impact on normal cells, but increases the effect of gefitinib. Once again gefitinib exposure results in a higher affinity, observed as a higher saturation of the receptors and, most of all, a slower dissociation.

The signal-to-noise ratio for the SKBR3 cells was the lowest of all cell lines due to the low number of EGFR in SKBR3 (Fig. 4D). This obstructed the analysis some, but we can conclude that there is no visible, statistical proven, effect on the ^125^I-EGF – EGFR interaction when the SKBR3 cells are exposed to either starvation or gefitinib. This is interesting as SKBR3 is the most sensitive to gefitinib of all four cell lines when studying growth rate inhibition [13].

All in all, gefitinib can affect the binding of ^125^I-EGF to EGFR in some cell lines. Sundberg et. al. [14] have previously shown that the uptake of ^125^I-EGF increases in the presence of 1 µM gefitinib for A431 and U343 cells. These studies were made at 37°C and one of the suggested explanations was that gefitinib affected the EGF internalization which in turn altered the uptake ability and the excretion rate of ^125^I bound to EGF. The link between internalization and gefitinib has been made by other groups as well [21], [22]. It is however possible that the amplified uptake signal for U343 and especially A431 as observed by Sundberg et al. [14] can be explained solely by an increased affinity, since the ^125^I-EGF concentration tested (2 nM) is close to K_D_ which makes the detected output highly sensitive to any affinity changes. The measurements in this paper were conducted at room temperature, and no signs of internalization were detected for the U343 cell line during a 24 hour timeframe (data not shown) implying that internalization can be neglected in all our experiments. Thus, gefitinib binding to the intracellular part of the EGF receptor can apparently change the extracellular binding properties of EGFR. If this effect is due to conformational changes of the receptor or intracellular processes is yet to be determined, although the increased effect of gefitinib in starved A431 and SKOV3 cells indicate that the condition of the cell is also important.

The surprisingly large difference in affinity of EGF-EGFR across different cell lines and treatments gives reason for reflection. Even though we have only investigated one ligand-receptor interaction in detail, we cannot exclude that the hosting cell line influences the interaction results also for other receptors. In our laboratory, we will continue to investigate various ligand-receptor interactions across different cell lines to better understand the importance of cellular context.

### Conclusions

By use of data from repeatable, high-precision interaction measurements, we conclude that at normal conditions, EGF interacts with EGFR in quite different manners depending on the hosting cell line. It is further evident that the addition of gefitinib or the lack of serum in the cell-culture medium can alter the EGF-EGFR interaction properties at least 10-fold, but again depending on the hosting cell line.

We have clear indications on that the apparent EGF-EGFR interaction is composed of multiple different interactions and that the affinity varies greatly between the cell lines, but more work is required to decipher the underlying nature of the EGF-EGFR interaction. Thus, the EGF receptor family, its multiple interactions with EGF and the effects of intracellular effects remains an unsolved puzzle with need for further investigation.

From a general perspective, it may be advisable to limit the discussion of any ligand-receptor interaction to the context of the hosting cell-line and care is recommended when generalizing interaction results across multiple cell-lines.

## Materials and Methods

### Cell culture

The human squamous carcinoma cell line A431 (CLR 1555, ATCC, Rocksville, MD, USA), the human glioma cell line U343MGaCl2:6 (denoted U343), the human ovarian carcinoma cell line SKOV3 (HTB-77, ATCC, Rocksville, MD, USA) and the human breast cancer cell line SKBR3 (HTB-30, ATCC, Rocksville, MD, USA) were used in the experiments. The cells were seeded on a local area of a cell culture dish (Nunclon™, Size 100×20, NUNC A/S, Roskilde, Denmark), as described previously [23]. Ham's F10 cell culture medium (Biochrom Ag, Berlin, Germany) supplemented with 10% fetal calf serum (Sigma, St Louis, MO, USA), L-glutamine (2 mM) and PEST (penicillin 100 IU/ml and streptomycin 100 µg/ml, from Biochrom Ag, Berlin Germany) was used if not otherwise specified. The cells were grown at 37°C in incubators with humidified air and 5% C0_2_.

### Radiolabeling

2.5 µg human Epidermal growth factor (EGF, Chemicon International, USA) was labeled at four occasions with 10–20 MBq ^125^I (Perkin-Elmer, Wellesley, MA, USA) using the Chloramine-T protocol [24]. Chloramine-T (Sigma, St Louis, MO, USA) and sodium metabisulfite (Aldrich, Stockholm, Sweden) were used for the labeling reactions. Excess reagents were separated from the protein using a NAP-5 column (GE Healthcare, Waukesha, WI, USA) equilibrated with PBS (10 mM, pH 7.4, 140 mM NaCl).

### Measurements of affinity in LigandTracer Grey

The binding of ^125^I-EGF to seeded cells were monitored in real-time at room temperature using LigandTracer Grey, as described preciously by Björke and Andersson [23]. Five increasing concentrations of ^125^I-EGF in complete medium was added in each affinity titration assay. The measurements were performed in duplicates for each cell line and different concentration series were used to fully cover the concentration span needed for a proper affinity estimation (Table 1). Each concentration was incubated long enough to approach steady state, from 1 hour for the highest concentrations to 3.5 hours for the lowest.

**Table 1:** Summary of concentrations tested.

	Affinity titration	Cell treatment assay	
Cell line	Concentration span	Conc. 1 (nM)	Conc. 2 (nM)
**A431**	0.3;1;3;9;28 nM	2.8	9
**A431**	3;9;36;72;138 nM		
**U343**	0.1;0.3;0.9;2.8;9.4 nM	0.5	1.5
**U343**	0.1;0.4;1.6;6.6;26 nM		
**SKOV3**	0.1;0.3;0.9;2.8 nM	0.7	2
**SKOV3**	0.05;0.15;0.5;1.5;4.6 nM		
**SKBR3**	0.3;1;3;9;28 nM	0.2	0.8
**SKBR3**	0.1;0.3;1;3;9 nM		

Concentrations of ^125^I-EGF tested on the four cell lines in the cell treatment assay and the affinity titration assay.

### Internalization measurement

U343 cells were incubated with 4.5 nM ^125^I-EGF in room temperature for 24 hours while detecting the ^125^I-EGF binding level in LigandTracer Grey. In case of internalization, EGF would be metabolized and free ^125^I would be excreted by the cells, resulting in a decreasing signal over time.

### Data analysis

The kinetic data from the affinity measurements were analyzed in TraceDrawer (Ridgeview Instruments AB, Uppsala, Sweden) and fitted to kinetic interaction models describing a monovalent ligand binding to either one (1∶1) target or two (1∶2) independent targets.

The estimated steady-state signals from both affinity measurements were analyzed simultaneously in MATLAB 6.5 (The Mathworks, Natick, MA, USA) and a 1∶1 steady state interaction model was fitted using non-linear regression to obtain the equilibrium dissociation constant, K_D_.

In order to enhance visibility of the acquired binding curves, a moving window Fourier transform noise reduction algorithm was applied. Noise with a frequency higher than approximately 0.01 Hz was removed, resulting in noise-reduced curves where the biologically relevant curvature was preserved.

### Cell treatments

Four kinds of treatment of the cells were tested: (a) in normal cell culture medium, as described above, (b) in cell culture medium devoid of fetal calf serum, (c) in cell culture medium supplemented with 1 µM gefitinib (Biaffin GmbH, Kassel, Germany) and (d) in cell culture medium devoid of serum but with 1 µM gefitinib. The cell culture medium of the starvation dishes (b) were exchanged with serum free Ham's F10 24 h prior to measurement. The gefitinib dishes (c) were incubated with 1 µM gefitinib 48 h before measurement. Fresh medium with 1 µM gefitinib was added after 24 h to ensure a continuous gefitinib exposure. Prior to retention measurements, more gefitinib was added to ensure a fresh gefitinib concentration of at least 1 µM. The gefitinib +starvation dishes (d) were treated similar to (c), but with serum free medium +1 µM gefitinib added 24 h prior to measurement.

### Measurements of cell treatment effects in LigandTracer Grey

Detections of treatment effects were performed at room temperature using LigandTracer Grey. All measurements were conducted in 3 ml cell culture medium and started with a short baseline, followed by a two-step uptake study using increasing concentrations of ^125^I-EGF (Table 1), followed by a retention measurement over night in cell culture medium. The incubation times were approximately 3.5+4 h for all cell lines. Each of the 16 cell-treatments was measured at least 2 times, often 3–4 times, resulting in mainly triplicate and quadruplicate results.
